# Life Course Socioeconomic Position and Mid-Late Life Cognitive Function in Eastern Europe

**DOI:** 10.1093/geronb/gbu014

**Published:** 2014-03-05

**Authors:** Pia Horvat, Marcus Richards, Sofia Malyutina, Andrzej Pajak, Ruzena Kubinova, Abdonas Tamosiunas, Hynek Pikhart, Anne Peasey, Michael G. Marmot, Martin Bobak

**Affiliations:** ^1^Department of Epidemiology and Public Health, University College London, UK.; ^2^Unit for Lifelong Health and Ageing, Medical Research Council, London, UK.; ^3^Department of Non-Invasive Diagnostics, Institute of Internal Medicine, Siberian Branch of the Russian Academy of Medical Sciences, Novosibirsk, Russia.; ^4^Department of Clinical Epidemiology and Population Studies, Jagiellonian University Medical College, Krakow, Poland.; ^5^Centre of Environmental Health, National Institute of Public Health, Prague, Czech Republic.; ^6^Department of Population Studies, Institute of Cardiology, Lithuanian University of Health Sciences, Kaunas, Lithuania.

**Keywords:** Cognitive aging, Eastern Europe, Life course, Socioeconomic position.

## Abstract

**Objectives.:**

To investigate whether the positive relation between socioeconomic position (SEP) across the life course and later life cognitive function observed in Western populations exists in former communist countries with apparently smaller income inequalities.

**Method.:**

Structural equation modeling analysis of cross-sectional data on 30,846 participants aged 45–78 years in four Central and Eastern European centers: Novosibirsk (Russia), Krakow (Poland), Kaunas (Lithuania), and six Czech towns from the HAPIEE (Health, Alcohol, and Psychosocial factors In Eastern Europe) study. SEP was measured using self-reported childhood (maternal education, household amenities), adult (education), and older adult (current material circumstances) indicators. Latent variable for cognition was constructed from word recall, animal naming, and letter search.

**Results.:**

Associations between SEP measures over the life course and cognition were similar across study centers. Education had the strongest direct association with cognition, followed by current material circumstances. Indirect path from education to cognition, mediated by current SEP, was small. Direct path from mother’s education to cognition was significant but modest, and partially mediated by later SEP measures, particularly education.

**Discussion.:**

In these Eastern European populations, late life cognition reflected life course socioeconomic trajectories similarly to findings in Western countries.

Cognitive aging represents a major societal burden and is associated with loss of independence (Greiner, Snowdon, & Schmitt, 1996), lower quality of life, and increased risk of premature mortality (Shipley, Der, Taylor, & Deary, 2006). Positive associations between socioeconomic position (SEP) across the life course and cognitive functioning in middle and older age (Haan, Al-Hazzouri, & Aiello, 2011; Luo & Waite, 2005; Richards & Sacker, 2003; Singh-Manoux, Richards, & Marmot, 2005; Turrell et al., 2002) highlight the public health importance of inequalities in this outcome in later life. The association between adult SEP and late life cognition may reflect the underlying social gradient in health, including life course history of chronic disease, access to health care, occupational and environmental exposures, chronic stress, and health-related behaviors.

Education and adult SEP are thought to benefit cognitive health in later life primarily by providing cognitive reserve, which in turn provides resilience to age-related neuropathology (Brayne et al., 2010). Education may directly influence cognition by promoting cognitive development through mental stimulation and enriched environments (Richards & Hatch, 2011), and greater occupational complexity may further increase cognitive performance (Hauser & Roan, 2007; Kohn & Schooler, 1978). Additionally, education may be indirectly linked to cognition by promoting attainment of higher status occupations and continued cognitive engagement. Finally, the association between education and cognition may, to some extent, reflect ability-based selection into education (Deary & Johnson, 2010). These processes may not be mutually exclusive, since education appears to augment cognition independently of prior cognitive ability (Clouston et al., 2012; Glymour, Kawachi, Jencks, & Berkman, 2008; Hatch, Feinstein, Link, Wadsworth, & Richards, 2007) and is a crucial mediator between cognitive ability and attained social position (Johnson, Brett, & Deary, 2010).

Increasingly, research on health in later life has come to adopt the life course approach (Kuh et al., 2012) whereby early childhood experiences may have long-lasting consequences for cognitive health (Whalley, Dick, & McNeill, 2006). Previously reported enduring effects of childhood SEP on cognition (Everson-Rose, de Leon, Bienias, Wilson, & Evans, 2003; Fors, Lennartsson, & Lundberg, 2009; Kaplan et al., 2001) could partly reflect the association of childhood SEP with early life cognitive development (Bradley & Corwyn, 2002), with plausible pathways including maternal exposures during gestation, maternal stress, nutrition, childhood health, parenting practices, mental stimulation, and childhood poverty. Early years are crucial for brain development, and it is conceivable that neurocognitive inefficiencies resulting from early life insults become exacerbated with cognitive aging (Moceri et al., 2001).

However, childhood SEP also has an important influence on future life chances. According to the cumulative advantage/disadvantage perspective (Dannefer, 1987), social inequalities in health are initiated early in life and increase with age as initial advantages or disadvantages accumulate and compound across the life course. In this way, childhood socioeconomic disadvantage restricts educational opportunities, which, in turn, limit employment options and negatively affect future income trajectories, leading to persisting inequalities (Ferraro & Kelley-Moore, 2003) or a divergence in health and well-being in later life.

In fact, in most cross-sectional studies (Fors et al., 2009; Haan et al., 2011; Luo & Waite, 2005; Singh-Manoux et al., 2005; Turrell et al., 2002) the association between childhood SEP and adult cognition was partially mediated through education and adult SEP. In longitudinal studies (Johnson, Gow, Corley, Starr, & Deary, 2010; Osler, Avlund, & Mortensen, 2013; Richards & Sacker, 2003), the association was fully or largely explained by childhood or adolescent cognitive ability and subsequent SEP. In addition, most of these life course studies also found strong independent associations between education and cognition, and significant but typically weaker associations between adult SEP and cognition. Notably, this pattern was reported by Richards and Sacker (2003) in the 1946 British birth cohort for measures of both crystalized and fluid cognition, and after adjusting for childhood cognitive ability. Thus, childhood SEP is associated with late life cognitive function, and this association appears to be mediated by cognitive development, education and adult SEP. In addition, each of these mediators is independently associated with cognitive aging in descending order of strength.

Most studies in this field come from Western populations. Although processes underlying associations between SEP and cognition may be largely universal, there is some evidence to suggest that the associations may be modified by contextual factors (Clouston et al., 2012; Glymour et al., 2008; Richards, Power, & Sacker, 2009). Richards et al. (2009) found that while the pattern of life course associations between SEP and adult literacy and numeracy was relatively stable across two U.K. birth cohorts, the strength of these associations varied, reflecting social changes that occurred in the postwar period. In a study of Chinese individuals social context also appeared to modify the association between childhood SEP and cognition across successive cohorts (Zhang et al., 2009).

The present study in four Central and Eastern European populations with historically smaller income inequalities adds a broader dimension to existing research on life course associations between SEP and cognition. In the second half of the 20th century, efforts of the Communist regimes to minimize material inequalities between different social groups resulted in relatively weak correlations between education or occupation and material circumstances (Connor, 1979; Flakierski, 1993). To the extent that differences in the socialist stratification pattern were consequential for life course accumulation of risk, this may be reflected in weaker associations between SEP and cognition in later life. In this study, we investigated the relationship between measures of socioeconomic position across the life course and cognitive function in midlife and early old age, using a large cross-sectional dataset from predominantly urban populations in the Czech Republic, Russia, Poland, and Lithuania. Structural equation modeling was used to examine the multiple pathways leading from life course SEP measures to cognition in these four populations.

## Method

### Study Populations and Participants

We used data from the HAPIEE (Health, Alcohol, and Psychosocial factors In Eastern Europe) study. Details of the study protocol were given elsewhere (Peasey et al., 2006). Briefly, random population samples of 28,945 men and women aged 45–69 at baseline in 2002–2005 were recruited from population registers in six Czech towns, Novosibirsk (Russia) and Krakow (Poland) with an average response rate of 59%. Most (18,154) participants were successfully reinterviewed between 2006 and 2008 with a response rate of 63% reflecting death and dropout. Another 7,164 participants from Kaunas (Lithuania) joined the study at that time with a response rate of 65%, giving a baseline study total of 36,109 participants. The study was approved by the ethical committee at University College London and in each participating center, and all participants gave informed consent to participate in the study.

In all countries participants completed an extensive health questionnaire, including a section on current and past socioeconomic circumstances, and underwent an examination in a clinic (Peasey et al., 2006). The baseline assessment of the three original cohorts involved testing of cognitive function in all retired participants and in an approximately 20% random subsample of working participants (different characteristics were studied in working and non-working participants but, for comparability, cognitive functions were also measured in a subsample of working persons). At reexamination 2–3 years later, cognitive function was assessed in all participants. In Lithuania cognitive function was assessed in all participants at baseline. Cognitive assessment was conducted as part of a clinic-based examination overseen by trained nurses, with the exception of participants in Poland, who were visited and examined in their homes. From 35,956 participants, 29,116 participants (74%, 81%, 77%, and 99% of all participants in Czech, Russian, Polish, and Lithuanian samples, respectively) completed at least one cognitive assessment; for those who completed both assessments, the first one was used. The follow-up questionnaire also included repeat measures of current socioeconomic circumstances which were used for participants whose first cognitive assessment was at follow-up.

### Cognitive Functions

Cognitive function was conceptualized as a latent variable using three measures of fluid cognitive ability. First, verbal memory was assessed using a word recall task consisting of 10 common nouns, administered over three consecutive 1-min trials. Word lists were the same in each country. Total number of words correctly recalled for each trial was summed to give an overall score (max. 30). Second, verbal fluency, a measure of executive function, was assessed by animal naming. Participants were asked to name as many different animals as possible within 1min. Names of different species counted toward the total but redundancies (e.g., black cow, brown cow) did not. Total number of correct responses was recorded. Third, mental speed and concentration was assessed using a paper and pencil letter cancellation task. Participants were instructed to cross out two target letters, P and W (P and Ш in Novosibirsk), randomly embedded within an A4 grid of other randomly chosen letters as quickly and as accurately as possible within 1min. Total number of letters correctly crossed out was used (max. 65).

### Indicators of SEP

Two childhood SEP indicators were used: (a) mother’s education was categorized into: less than primary, primary, secondary, and university; (b) access to any of the following basic household amenities when participants were aged approximately 10 years: cold and hot tap water, radio, fridge, own kitchen, and own toilet (range 0–6). These measures were collected at survey baseline. In the Czech study, questions on parental education were not included at baseline but were available at follow-up, and we used information on parental education from follow-up; given attrition between baseline and follow-up, 16% of Czech participants with data on cognition had missing values for parental education.

Own education was categorized into three groups: primary or less, secondary, and university. Adult SEP was represented by the number of household assets (car, mobile phone, color TV, satellite/cable TV, video recorder, video camera, fridge, microwave, dishwasher, washing machine, and landline) owned at cognitive assessment (range 0–11).

### Statistical Analysis

**Figure 1.**
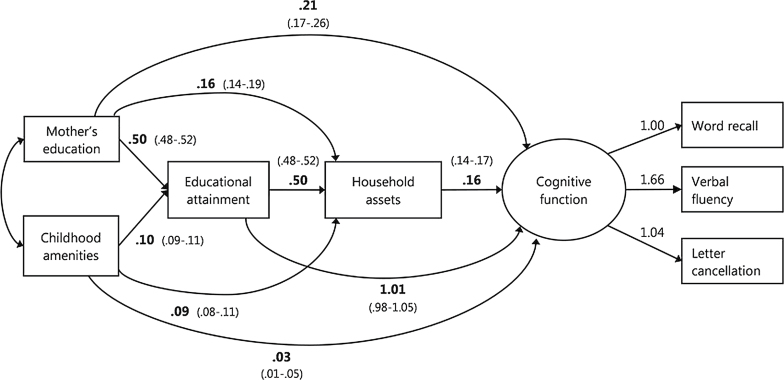
Unstandardized regression estimates with 95% confidence intervals from the multiple-group structural equation model with all structural parameters constrained across groups representing pathways between childhood SEP, educational attainment, adult household assets, and cognitive function (*n* = 30,846; *χ* ^2^ = 1,326.335, df = 178; comparative fit index [CFI] = 0.954; Tucker–Lewis index [TLI] = 0.949; root mean square error of approximation [RMSEA] = 0.041 [0.039–0.043]).

The outcome variable was latent cognitive function construct; confirmatory factor analysis showed substantial common variance shared by the different cognitive measures (verbal memory, verbal fluency, and mental speed) thought to reflect a general cognitive factor. Structural equation modeling was used to examine multiple pathways linking life course SEP measures to mid and late life cognitive function. In the structural model, education and household assets were assumed to partially mediate the associations of childhood SEP with cognition, while direct paths from childhood SEP measures to the latent cognitive factor were also estimated (see Figure 1). Education was entered as a categorical mediating variable. The model was adjusted for age at cognitive assessment, assuming direct paths leading from age to cognition, assets, and education. The model was estimated conditional on covariances between observed exogenous variables, which include childhood SEP measures and age.

Model fit was assessed using the comparative fit index (CFI), Tucker–Lewis index (TLI), and root mean square error of approximation (RMSEA). CFI and TLI values >0.90 and >0.95 indicate acceptable and good fit, and RMSEA values <0.05 indicate good fit. We did not use *χ* ^2^ statistic as it is overly sensitive to model misspecification with large sample sizes. Model estimation employed the robust (mean and variance adjusted) weighted least squares (WLSMV) estimator for categorical mediators, which handles missing data using pairwise present (Asparouhov & Muthén, 2010). We compared results after listwise deletion on all variables in the model (*n* = 25,127) to those obtained with pairwise present (*n* = 30,846). As preliminary analyses and previous research suggested a possible modifying role of study center and gender, a multiple-group model was specified (center × gender).

Measurement invariance of the latent cognitive factor was assessed by first fitting the measurement model separately in all groups. Second, multiple-group analysis was used to specify a gradually more restrictive model by sequentially constraining factor loadings, and intercepts across groups, and, finally, by adding covariates. These analyses were repeated for the eight-group model.

We first estimated the full structural equation model separately in each group. This was followed by a multiple-group model in all eight groups. Multiple-group model with all structural parameters constrained was used to obtain estimates averaged across groups. To explore differences between groups we compared this model to the one where all structural parameters were freely estimated in each group. Paths that differed significantly between groups were identified by post hoc analyses of model fit. Data preparation and descriptive analyses were conducted in Stata 12 (StataCorp, 2011), and all structural equation analyses were conducted in Mplus 6.12 (Muthén & Muthén, 1998-2011).

## Results

Summary statistics and frequency distributions of the study variables are presented in Table 1. Average age of participants was 60 years, and 55% of participants were female. Russians had lower means of childhood and current material circumstances than the other study centers (both *p* values *p* < .001, Bonferroni adjusted), consistent with generally poorer socioeconomic conditions in Russia. Participants’ level of education exceeded that of their mothers.

**Table 1.** Descriptive Characteristics of Study Sample (Based on Listwise Deletion, *n* = 25,127)

	Men				Women			
	Czech Republic (*n* = 2,203)	Russia (*n* = 2,747)	Poland (*n* = 3,661)	Lithuania (*n* = 2,966)	Czech Republic (*n* = 2,603)	Russia (*n* = 3,421)	Poland (*n* = 3,890)	Lithuania (*n* = 3,636)
Mean word recall	21.9 (3.5)	20.6 (4.6)	19.9 (4.2)	20.9 (4.1)	23.5 (3.4)	21.8 (4.3)	21.0 (4.2)	22.7 (3.7)
Mean verbal fluency	24.1 (6.7)	19.4 (7.2)	21.0 (6.4)	21.6 (6.0)	24.2 (6.3)	19.1 (6.9)	21.0 (6.3)	21.5 (6.1)
Mean letter cancellation	17.5 (4.6)	16.7 (5.1)	17.5 (5.7)	15.5 (4.6)	18.6 (4.6)	18.1 (5.4)	18.4 (5.9)	16.8 (4.8)
Mean age	60.4 (6.5)	59.4 (6.4)	60.5 (5.5)	60.5 (7.6)	59.5 (6.4)	59.2 (6.4)	59.9 (5.7)	60.2 (7.6)
Mean childhood amenities	4.0 (1.4)	2.2 (1.7)	3.2 (1.9)	3.0 (1.3)	4.1 (1.4)	2.3 (1.6)	3.3 (1.9)	3.0 (1.3)
Mother’s education, %								
Less than primary	2.2	19.4	11.0	17.4	2.7	21.4	10.2	19.6
Primary	51.2	33.6	52.3	55.7	50.3	32.9	53.0	55.1
Secondary	44.9	41.2	32.8	21.7	46.0	41.1	32.5	21.2
University	1.6	5.9	4.0	5.2	1.0	4.6	4.2	4.0
Education, %								
Primary	3.7	10.5	9.1	12.2	14.5	8.7	13.7	10.6
Secondary	74.4	54.5	59.1	52.8	73.8	62.4	58.9	56.1
University	21.9	34.9	31.9	35.0	11.8	28.9	27.4	33.3
Mean household assets	7.1 (1.9)	5.7 (2.1)	6.6 (2.1)	7.1 (2.0)	6.6 (1.9)	5.3 (2.1)	6.0 (2.1)	6.3 (2.0)

*Note.* Standard deviations in parentheses.

The correlations between different cognitive tests were similar across study centers (see Supplementary Table 1) and ranged from 0.28 (*p* < .001) between verbal memory and letter search in Czechs to 0.54 (*p* < .001) for verbal fluency and verbal memory in Poles. Measurement invariance testing provided support for invariance of factor loadings but not invariance of intercepts across groups (further details are given in Supplementary Table 2). Invariance of factor loadings is sufficient for comparison of structural parameters across groups (Byrne, Shavelson, & Muthen, 1989).

Results for the structural equation model were estimated both for complete cases (listwise deletion) and after including missing data (pairwise present with WLSMV estimation). The results were very similar; therefore only results from the latter are reported. Estimates for the model with all structural paths constrained across groups are reported in Figure 1. Unstandardized estimates are preferred for comparing groups because different variances between groups may lead to different standardized estimates even with the same unstandardized solution.

The fully constrained multiple-group model had an adequate fit to the data (*χ* ^2^ = 1,326.3, *df* = 178; CFI = 0.954; TLI = 0.949; RMSEA = 0.041 [0.039–0.043]). This model revealed that SEP measures from all stages of the life course were significantly (*p* < .001) associated with cognition in mid and later life. Only childhood amenities were not substantively associated with cognition (*p* = .013).

Among the life course SEP measures the strongest direct path was from own education to cognition; indirect effect of education through current asset ownership was small. The path from education was followed by a weaker path from household asset ownership, a measure of current SEP. Although statistically significant, the direct path from mother’s education to the latent cognitive factor was weak. Additionally, mother’s education had a significant (*p* < .001) indirect effect on cognition, largely mediated by participants’ education. The indirect effect of mother’s education was greater than its direct effect. The indirect effect of childhood amenities on cognition was very small.

Estimates for the unconstrained model are reported in Tables 2 and 3 for men and women, respectively. The unconstrained model was a significant improvement over the fully constrained model (*χ* ^2^ = 621.8, df = 94; CFI = 0.979; TLI = 0.955; RMSEA = 0.038 [0.035–0.041]). Post hoc evaluation of model fit identified parameters that differed significantly across groups.

**Table 2.** Results of the Unconstrained Multiple-Group Structural Equation Model for Men

	Czech Republic (*n* = 2,499)				Russia (*n* = 3,789)				Poland (*n* = 5,036)				Lithuania (*n* = 3,054)			
	Est	*SE*	*p* Value	Std	Est	*SE*	*p* Value	Std	Est	*SE*	*p* Value	Std	Est	*SE*	*p* Value	Std
Direct effects on cognition																
Mother’s education	0.49	0.11	<.001	0.12	0.12	0.08	.112	0.03	0.26	0.08	.001	0.06	0.14	0.08	.063	0.04
Childhood amenities	−0.02	0.05	.697	−0.01	0.05	0.04	.236	0.03	0.08	0.03	.016	0.05	−0.07	0.05	.120	−0.04
Education	0.94	0.07	<.001	0.42	0.82	0.07	<.001	0.29	0.94	0.06	<.001	0.36	1.13	0.06	<.001	0.50
Household assets	0.09	0.03	.003	0.08	0.30	0.03	<.001	0.21	0.24	0.03	<.001	0.17	0.11	0.03	<.001	0.09
Direct effects on household assets																
Mother’s education	0.23	0.07	.001	0.07	0.13	0.05	.006	0.06	0.17	0.05	.001	0.06	0.07	0.05	.170	0.03
Childhood amenities	0.10	0.03	.001	0.07	0.03	0.02	.281	0.02	0.09	0.02	<.001	0.08	0.10	0.03	.002	0.06
Education	0.46	0.05	<.001	0.26	0.51	0.04	<.001	0.27	0.65	0.04	<.001	0.34	0.50	0.04	<.001	0.28
Direct effects on education																
Mother’s education	0.41	0.05	<.001	0.22	0.41	0.03	<.001	0.33	0.52	0.03	<.001	0.34	0.54	0.03	<.001	0.37
Childhood amenities	0.11	0.02	<.001	0.15	0.03	0.02	.086	0.04	0.12	0.01	<.001	0.20	0.05	0.02	.015	0.06
Indirect effects on cognition																
Mother’s education → education	0.41	0.05	<.001	0.10	0.38	0.04	<.001	0.11	0.50	0.04	<.001	0.12	0.61	0.05	<.001	0.18
Mother’s education → assets	0.02	0.01	.034	0.01	0.05	0.01	<.001	0.01	0.03	0.01	.016	0.01	0.01	0.01	.159	0.00
Mother’s education → education → assets	0.02	0.01	.005	0.01	0.07	0.01	<.001	0.02	0.09	0.01	<.001	0.02	0.03	0.01	<.001	0.01
Total indirect	0.45	0.06	<.001	0.11	0.50	0.04	<.001	0.14	0.62	0.04	<.001	0.15	0.65	0.05	<.001	0.20
Amenities → education	0.11	0.02	<.001	0.06	0.03	0.01	.007	0.02	0.12	0.01	<.001	0.07	0.06	0.02	.011	0.03
Amenities → assets	0.01	0.00	.025	0.01	0.01	0.01	.068	0.01	0.02	0.01	<.001	0.02	0.01	0.00	.013	0.01
Amenities → education → assets	0.01	0.00	.009	0.00	0.01	0.00	.009	0.00	0.02	0.00	<.001	0.01	0.00	0.00	.028	0.00
Total indirect	0.12	0.02	<.001	0.07	0.05	0.01	.001	0.03	0.16	0.01	<.001	0.10	0.08	0.03	.003	0.04
Education → assets	0.04	0.02	.003	0.02	0.15	0.02	<.001	0.06	0.17	0.02	<.001	0.06	0.06	0.01	<.001	0.03
Total effects on cognition																
Mother’s education	0.94	0.11	<.001	0.23	0.56	0.07	<.001	0.16	0.93	0.08	<.001	0.22	0.78	0.07	<.001	0.24
Childhood amenities	0.10	0.05	.036	0.06	0.09	0.04	.018	0.05	0.24	0.03	<.001	0.15	0.00	0.05	.980	0.00
Education	0.98	0.07	<.001	0.44	1.00	0.06	<.001	0.36	1.11	0.06	<.001	0.41	1.21	0.06	<.001	0.53

*Note.* Fit indices: *χ* ^2^ (94) = 621.824; comparative fit index = 0.979; Tucker–Lewis index = 0.955; root mean square error of approximation = 0.038 (0.035–0.041). Paths from mother’s education and childhood amenities to education are probit coefficients. Est = unstandardized path coefficient; Std = standardized path coefficient.

**Table 3.** Results of the Unconstrained Multiple-Group Structural Equation Model for Women

	Czech Republic (*n* = 2,991)				Russia (*n* = 4,488)				Poland (*n* = 5,281)				Lithuania (*n* = 3,708)			
	Est	*SE*	*p* Value	Std	Est	*SE*	*p* Value	Std	Est	*SE*	*p* Value	Std	Est	*SE*	*p* Value	Std
Direct effects on cognition																
Mother’s education	0.42	0.10	<.001	0.11	0.20	0.07	.002	0.06	0.30	0.08	<.001	0.07	0.02	0.07	.743	0.01
Childhood amenities	−0.07	0.05	.113	−0.04	0.12	0.03	<.001	0.07	0.05	0.03	.130	0.03	−0.06	0.04	.117	−0.03
Education	1.05	0.06	<.001	0.52	0.81	0.06	<.001	0.32	1.05	0.06	<.001	0.40	1.19	0.05	<.001	0.54
Household assets	0.04	0.03	.132	0.04	0.26	0.03	<.001	0.19	0.10	0.03	<.001	0.07	0.10	0.02	<.001	0.08
Direct effects on household assets																
Mother’s education	0.21	0.07	.001	0.06	0.21	0.04	<.001	0.08	0.12	0.05	.015	0.04	0.19	0.05	<.001	0.07
Childhood amenities	0.09	0.03	.004	0.06	0.08	0.02	<.001	0.07	0.10	0.02	<.001	0.09	0.14	0.03	<.001	0.09
Education	0.41	0.04	<.001	0.23	0.38	0.04	<.001	0.20	0.52	0.04	<.001	0.29	0.40	0.04	<.001	0.22
Direct effects on education																
Mother’s education	0.51	0.04	<.001	0.27	0.45	0.03	<.001	0.35	0.57	0.03	<.001	0.35	0.51	0.03	<.001	0.34
Childhood amenities	0.15	0.02	<.001	0.19	0.07	0.02	<.001	0.10	0.15	0.01	<.001	0.24	0.09	0.02	<.001	0.10
Indirect effects on cognition																
Mother’s education → education	0.55	0.05	<.001	0.14	0.36	0.03	<.001	0.11	0.61	0.04	<.001	0.14	0.62	0.04	<.001	0.19
Mother’s education → assets	0.01	0.01	.179	0.00	0.06	0.01	<.001	0.02	0.01	0.01	.036	0.00	0.02	0.01	.008	0.01
Mother’s education → education → assets	0.01	0.01	.118	0.00	0.05	0.01	<.001	0.01	0.03	0.01	<.001	0.01	0.02	0.01	<.001	0.01
Total indirect	0.57	0.05	<.001	0.15	0.47	0.03	<.001	0.14	0.65	0.04	<.001	0.15	0.66	0.04	<.001	0.20
Amenities → education	0.16	0.02	<.001	0.10	0.05	0.01	<.001	0.03	0.01	0.00	.001	0.01	0.11	0.02	<.001	0.05
Amenities → assets	0.00	0.00	.168	0.00	0.02	0.01	<.001	0.01	0.01	0.00	<.001	0.01	0.01	0.00	.002	0.01
Amenities → education → assets	0.00	0.00	.121	0.00	0.01	0.00	<.001	0.00	0.05	0.03	.130	0.03	0.00	0.00	.002	0.00
Total indirect	0.17	0.02	<.001	0.10	0.07	0.01	<.001	0.04	0.18	0.01	<.001	0.11	0.13	0.02	<.001	0.06
Education → assets	0.02	0.01	.115	0.01	0.10	0.01	<.001	0.04	0.06	0.01	<.001	0.02	0.04	0.01	<.001	0.02
Total effects on cognition																
Mother’s education	1.00	0.09	<.001	0.26	0.65	0.06	<.001	0.19	0.95	0.08	<.001	0.22	0.70	0.07	<.001	0.21
Childhood amenities	0.12	0.04	.008	0.07	0.22	0.03	<.001	0.13	0.23	0.03	<.001	0.14	0.06	0.04	.160	0.03
Education	1.06	0.06	<.001	0.52	0.92	0.06	<.001	0.35	1.11	0.06	<.001	0.42	1.26	0.05	<.001	0.56

*Note.* Fit indices: *χ* ^2^ (94) = 621.824; comparative fit index = 0.979; Tucker–Lewis index = 0.955; root mean square error of approximation = 0.038 (0.035–0.041). Paths from mother’s education and childhood amenities to education are probit coefficients. Est = unstandardized path coefficient; Std = standardized path coefficient.

The path from assets to cognition was stronger in Poland and, especially, Russia than in Lithuania and the Czech Republic. Asset ownership tended to be more strongly associated with cognition in men than in women, especially in Polish and Czech samples. In Russian women, the path from childhood amenities to cognition was statistically significant and at least as important as the path from mother’s education. Mother’s education was more strongly associated with cognition in Czechs compared with other centers. The path from education was the weakest in Russians and the strongest in Lithuanians, and both were significantly different from the average effect of education estimated in the fully constrained model.

When the analysis was repeated using father’s education the pattern of results was similar but mother’s education was generally more strongly associated with cognition (see Supplementary Table 3).

Analyses of SEP at different stages of the life course revealed the following pattern. First, own education was significantly influenced by mother’s education. Second, childhood amenities had a small direct effect on own education, and third, current household asset ownership received small direct inputs from childhood SEP measures and a moderate input from own education. Results were similar across study centers.

The structural equation analysis, repeated with the three cognitive measures as outcomes, is shown in Supplementary Table 4. The results were similar to those observed for the latent cognitive factor. The strongest path to each cognitive outcome was from education, followed by a weaker path from household assets. Modest significant paths were observed from mother’s education to verbal cognitive measures, while its association with letter cancellation was less consistent and on average stronger in men. Childhood amenities showed a significant but only moderately strong direct path to verbal memory only in Russians.

## Discussion

This study in four Central and Eastern European population samples suggests that in these populations cognitive function in mid and later life reflects the influence of SEP at several stages of the life course (childhood, adulthood, and middle-older age), similarly to studies in the West. We found a strong association of education with cognition, a weaker path from current SEP (approximated by household asset ownership), and a generally significant but modest direct path from mother’s education to cognition. A considerable proportion of the total effect of mother’s education on cognition was indirect, mediated by participants’ education, and, to a lesser degree, current asset ownership. In contrast, education had only a very small indirect effect on cognition transmitted through household assets. The pattern of results was broadly similar, albeit with some differences, across study centers.

The results of this study should be interpreted in the context of its limitations. First, the study had a cross-sectional design and childhood SEP measures were reported retrospectively. We cannot rule out the possibility that reporting accuracy is affected by cognitive status. Retrospective reports are also vulnerable to misclassification and/or bias, and this could have led to underestimation of associations between childhood SEP and cognition. However, simple sociodemographic information appears to be recalled reasonably accurately even in old age (Berney & Blane, 1997). Our additional sensitivity analyses (not shown in tables) confirmed that measurement error would not change the conclusions regarding the role of childhood SEP measures and current household assets. In addition, adjustment for misclassiﬁcations in predictor variables with relatively few categories is likely to have only a limited effect on coefficient sizes (Goldstein, Kounali, & Robinson, 2008).

Second, with the exception of Lithuania, we did not have complete cognitive data collected at baseline on all participants, and participants who did not attend the reexamination had lower baseline cognitive scores. Participants with missing data on cognitive function were younger, more likely to be male, had lower educational attainment, higher childhood SEP, and owned fewer assets than those with cognitive data, but this is unlikely to bias the estimates of the associations between SEP and cognition. Respondents with lower scores on SEP measures had a higher chance of attrition. However, additional analyses in participants who had baseline cognitive data but were lost to follow-up gave similar results to estimates from participants who remained in the study. Attrition is therefore unlikely to introduce a major bias.

Third, our results pertain to specific urban populations, not countries. However, levels and trends in socioeconomic indicators and mortality in the participating towns and cities are similar to their respective countries, and it is very likely that these study samples broadly reflect the situation in their countries’ urban populations.

Fourth, another important limitation associated with cross-sectional design is lack of a measure of prior cognitive ability, raising the challenge of reverse causality. Two studies (Osler et al., 2013; Richards & Sacker, 2003) that were able to adjust for childhood or adolescent cognitive ability observed significant independent effects of education and adult social class, and a fully or largely indirect effect of childhood SEP on midlife cognition; in another study, only childhood cognitive ability and education were associated with cognition in old age (Johnson, Gow, et al., 2010). Thus, we may have overestimated the structural effects of life course SEP on cognition. In addition, we had no relevant data to investigate the issue of reverse causality, although both social selection and causation mechanisms are likely to be important (Clouston et al., 2012).

Finally, estimation of direct and indirect effects in mediation analysis assumes there is no unmeasured confounding of the exposure–outcome, mediator–outcome, and exposure–mediator relationships (VanderWeele, 2010). Specification of the model and selection of covariates was based on substantive knowledge but residual confounding remains a possibility. Thus, any conclusions should be drawn cautiously.

Despite the limitations, this study is the first of its kind in the region where individual-level data are still not widely available. We were able to take advantage of a large multicenter cohort with objective verbal and non-verbal neuropsychological measures and relatively good data on SEP. Several findings deserve a comment.

The path from education to cognition was consistently strong, especially relative to other SEP measures, confirming the education–cognition relationship found previously (Cagney & Lauderdale, 2002; Lee, Buring, Cook, & Grodstein, 2006). This may owe to mental stimulation provided by education with potentially lasting benefits for cognitive reserve. Beyond direct influences on cognition, education is a significant determinant of occupational status with implications for cognitive aging. This aspect was especially important in credential-based labor markets typical of communist societies. Educational qualifications were the main basis for labor allocation and the main route to professional occupations, resulting in stronger associations between education and occupation than in Western countries (Titma, Tuma, & Roosma, 2003). However, education was only weakly correlated with higher incomes, and material inequalities were probably less important in mediating the association between education and cognitive health.

Another plausible contributing explanation for the strong education–cognition link in these societies is related to communist regimes’ efforts to create a greater equality of educational opportunity, especially in favor of children from disadvantaged socioeconomic backgrounds. Consequently, ability-based selection into education may have increased in importance relative to family background (Heath et al., 1985), resulting in a high correlation between educational attainment and cognitive ability.

Some reductions in social origin-based educational inequality were mostly achieved, particularly in the immediate postwar period. Despite this, parental background remained a significant determinant of offspring’s educational chances throughout the period, possibly even stronger than in Western countries (Ganzeboom & Nieuwbeerta, 1999; Nieuwbeerta & Rijken, 1996). Educational expansion and industrialization rather than egalitarian policies were the most significant forces shaping educational inequalities over this period (Nieuwbeerta & Rijken, 1996; Simonova, 2008). These trends were common to all Central and Eastern European countries but were modified by national historic and institutional contexts. Central European countries inherited educational systems with early tracking and a stronger vocational orientation in secondary education which, compared with the Soviet Union, may lead to greater inequality (Noelke, Kogan, & Gebel, 2012). Moreover, egalitarian policies, especially those directly aimed at improving educational access of children from disadvantaged backgrounds, were not always systematically pursued. In Soviet Russia, pressures created by the rapid expansion of secondary schooling inadvertently increased inequality in access to higher education (Gerber & Hout, 1995). In our study, the differences in magnitude of the education–cognition association, measured as unstandardized path coefficients, ranged from 0.81 in Novosibirsk (Russia) to 1.19 in Kaunas (Lithuania) and this statistically significant variation may be partly explained by cross-country differences in contextual factors.

We observed a generally significant effect of household assets currently owned, particularly in Russia and Poland, pointing to the potential importance of material circumstances for mid–late life cognition in these countries. Previous studies have reported an independent association of cognition with income and wealth (Cagney & Lauderdale, 2002; Lee et al., 2006). In post-Soviet Russia, with widening income (Milanovicć, 1998) and health (Shkolnikov et al., 2006) inequalities, material circumstances may have become more prominent in determination of health compared with other post-communist countries, such as the Czech Republic, where this trend has been less apparent.

Mother’s education showed a significant direct path to cognition in all but one study center. Previous research also found a stronger association between mother’s education and mid or late life cognition compared with paternal measures (Kaplan et al., 2001; Luo & Waite, 2005). Given the rapid educational expansion and removal of gender inequalities in education under socialism (Ganzeboom & Nieuwbeerta, 1999), rising levels of women’s education over the period may have a positive impact on cognitive aging not just in the more recent cohorts of women but also in the future generations of men and women.

On the other hand, significant path from childhood amenities to cognition was observed only in Russian women. The latter observation might reflect the greater degree of material disadvantage experienced by Russian cohorts in childhood (Haynes & Husan, 2003). Childhood conditions were found to be strongly associated with cognitive impairment in oldest old Chinese, where severe childhood adversity was common (Zhang, Gu, & Hayward, 2008). It has previously been found that Russians who were born before or during World War II were shorter than those born later, after controlling for secular trend (Webb et al., 2008). It is plausible that childhood conditions of these participants affected not only their height but also their cognition in later life, although in the latter case the effect was only salient in women.

Consistent with other work we observed significant tracking of SEP over the life course in these post-communist countries. Mother’s (and father’s) education had a notable influence on participants’ educational attainment, whereas the effect of childhood material conditions was less important. In societies where opportunities for accumulation and intergenerational transmission of wealth were limited, family cultural capital embodied in parents’ education may have been especially important for offspring’s educational attainment (Mateju & Rehakova, 1996). Both childhood SEP measures also had small independent effects on current material circumstances.

In the light of increasing economic returns to education reported in the region since the onset of transition (Brainerd, 1998), the finding of a moderate effect of education on current asset ownership in these relatively recent (post-1990) data may be important. These paths, particularly those involving participants’ education, were important in mediating the associations between parental education and childhood material conditions and cognition later in life. This indicates some degree of accumulation of childhood social disadvantage and advantage on cognitive function across the life course, consistent with previous studies showing considerable tracking of social disadvantage/advantage, with a cumulative effect on self-rated health (Nicholson, Bobak, Murphy, Rose, & Marmot, 2005) and depression (Nicholson et al., 2008). However, these findings are based on data collected after the fall of communism and, to some extent, probably also partly reflect the effects of economic transition. At least in Russia, the association between parental and adult socioeconomic position may have actually strengthened as a result of economic transition (Gerber & Hout, 2004).

Although the level of inequality under communism remains a matter of debate, Czechoslovakia is generally considered to have been the most equal among Central and Eastern European communist countries in terms of both wage and income inequality (Atkinson & Micklewright, 1992; Flemming & Micklewright, 2000). In comparison, inequalities are believed to have been greater in the former Soviet Union republics, with communist Poland falling somewhere in-between. In the post-communist period increase in income inequality has been relatively slow and contained in the Czech Republic, somewhat greater and quicker in Poland and rapid and dramatic in the Russian Federation, with Lithuania falling somewhere in-between the latter two countries (Bandelj & Mahutga, 2010; Heyns, 2005).

The general pattern of results on the relationship between SEP and cognitive function in our study does not unequivocally support the hypothesis that the overall level of social inequality (measured by indicators such as income inequality) in a society is positively associated with greater life course inequalities in cognitive outcomes at the individual level, at least not within Central and Eastern Europe or with respect to all SEP measures. In our study, this hypothesis was partly sustained with respect to current material circumstances, which showed a stronger association with cognitive function in the Polish and, especially, Russian samples than in the Czech sample, consistent with the level of income inequality in these countries. The results for Lithuania do not conform to the expected pattern but this may partly reflect the nature of more recently collected data, as the relatively basic items used to construct the asset index may have become increasingly common and less informative in the more recent period.

In contrast to current material circumstances, the strongest associations between education and cognitive function were observed in Lithuania and the weakest in Russia, whereas associations in the Czech Republic and Poland were of similar magnitude. Similarly, the association between mother’s (and father’s) education and cognitive function was generally strongest in the Czech sample (Czech and Polish samples for father’s education) and insignificant in Lithuania. Thus, the pattern of results for own or parental education and cognition does not conform to any simple expectation about how the education–cognition link may be modified by the different levels of inequality between these countries.

However, social inequalities in education may be more closely linked to the level of modernization and organization of the school system (e.g., tracking and selection) than to the overall level of societal inequality (Marks, 2005), and it is possible that the education–cognition link is affected by similar forces. In addition, although the differences in the magnitude of associations between countries may reflect genuine contextual differences, they may be due to chance.

Despite differences in methodology, comparing our results with studies in Western populations may still be useful. Qualitative comparisons suggest that in our study associations between life course SEP measures and cognition were either within the range observed in studies of Western populations or stronger. For example, in our study standardized effect sizes for education–cognition association were generally moderate and ranged from 0.29 to 0.52 for the latent cognitive trait, 0.17 to 0.34 for word recall, 0.16 to 0.34 for letter cancellation, and 0.18 to 0.37 for verbal fluency (see Supplementary Table 4) across populations. In comparison, education–cognition association was weaker in the 1921 Scottish birth cohort (0.08 for age 70 IQ) (Johnson, Gow, et al., 2010), weaker or within range in the 1946 British birth cohort (standardized coefficients were 0.22 for word recall, 0.08 for letter cancellation, and 0.24 for crystalized intelligence) (Richards & Sacker, 2003) and a Chicago-based aging study (0.16 for global cognition) (Jefferson et al., 2011), and within range in the Health and Retirement Study (≈0.36 for global cognition) (Luo & Waite, 2005). Findings for the associations between adult SEP or childhood SEP measures and cognition were generally similar.

However, several aforementioned (Jefferson et al., 2011; Johnson, Gow, et al., 2010; Richards & Sacker, 2003) studies in Western populations controlled for additional factors, most notably childhood cognitive ability or premorbid intelligence, that we were unable to control for. Adjustment for these factors is expected to weaken the direct associations between SEP measures and cognitive function. The observed differences in the magnitude of effects between studies may partly reflect incomplete adjustment in our study, as well as other differences in study design. However, associations between the different SEP measures were also of similar magnitude or stronger than in studies of Western populations, suggesting that in these Central and Eastern European populations tracking of social disadvantage/advantage across the life course was no less significant for cognitive outcomes in middle and older age than in the West.

Emerging evidence of broadly similar patterns of associations between life course SEP and cognition in mid-later life found across different settings, including the four Central and Eastern European populations in this study, and settings as diverse as the United States (Luo & Waite, 2005), Great Britain (Richards & Sacker, 2003), Scandinavia (Fors et al., 2009; Osler et al., 2013), and China (Zhang et al., 2009) suggests that the mechanisms underlying the associations may be pervasive. In addition, the findings also suggest that the fundamentally different economic structure of the communist system, in the several decades of its existence, did not necessarily result in significantly different patterns of health inequalities from contemporary capitalist societies.

In the Warsaw study of children born in the 1960s, the association between parental social background and childhood cognitive ability was, contrary to authors’ expectations, at least as strong, if not stronger, as in the West, despite the relative absence of educational, health care, and community divisions typically associated with socioeconomic position (Firkowska et al., 1978). Our study, in four urban populations in Central and Eastern Europe, confirms that the influence of life course socioeconomic trajectory is still reflected in cognitive function in midlife and beyond, similarly to Western populations. This suggests a largely universal structure of associations between SEP across the life course and cognition in later life.

## Supplementary Material

Supplementary material can be found at: http://psychsocgerontology.oxfordjournals.org/

## Funding

This study was supported by the Wellcome Trust (grant numbers WT0 064947, WT0 81081); the US National Institute of Aging (grant number R01 AG23522); the Medical Research Council funding to M. Richards; and the Economic and Social Research Council funding to P. Horvat.
